# Microarray Analysis of Transcriptome of Medulla Identifies Potential Biomarkers for Parkinson's Disease

**DOI:** 10.1155/2013/606919

**Published:** 2013-11-20

**Authors:** Xiao-Yang Liao, Wei-Wen Wang, Zheng-Hui Yang, Jun Wang, Hang Lin, Qing-Song Wang, Yu-Xian Wu, Yu Liu

**Affiliations:** ^1^Unit of General Practice, West China Hospital of Sichuan University, Chengdu 610041, China; ^2^Department of Neurology, Cheng Du Military General Hospital, Chengdu 610083, China

## Abstract

To complement the molecular pathways contributing to Parkinson's disease (PD) and identify potential biomarkers, gene expression profiles of two regions of the medulla were compared between PD patients and control. GSE19587 containing two groups of gene expression profiles [6 dorsal motor nucleus of the vagus (DMNV) samples from PD patients and 5 from controls, 6 inferior olivary nucleus (ION) samples from PD patients and 5 from controls] was downloaded from Gene Expression Omnibus. As a result, a total of 1569 and 1647 differentially expressed genes (DEGs) were, respectively, screened in DMNV and ION with limma package of *R*. The functional enrichment analysis by DAVID server (the Database for Annotation, Visualization and Integrated Discovery) indicated that the above DEGs may be involved in the following processes, such as regulation of cell proliferation, positive regulation of macromolecule metabolic process, and regulation of apoptosis. Further analysis showed that there were 365 common DEGs presented in both regions (DMNV and ION), which may be further regulated by eight clusters of microRNAs retrieved with WebGestalt. The genes in the common DEGs-miRNAs regulatory network were enriched in regulation of apoptosis process via DAVID analysis. These findings could not only advance the understandings about the pathogenesis of PD, but also suggest potential biomarkers for this disease.

## 1. Introduction

Parkinson's disease (PD) is the second most common neurodegenerative disorder in human, which is characterized by progressive death of dopamine-generating cells in the substantia nigra and accumulation of intraneuronal Lewy bodies containing misfolded fibrillar *α*-synuclein (SNCA), which eventually results in progressive movement disorders, including shaking, rigidity, bradykinesia, and gait disturbance [1]. Epidemiologic studies have identified environmental factors such as trauma [2] and pesticide exposure [3, 4] as risk factors for PD, while the increasing evidence demonstrates that genetic factors play significant roles in PD. Several genes have been linked to PD, such as SNCA, leucine-rich repeat kinase 2 (LRRK2), parkin (PARK2), PTEN-induced kinase 1 (PINK1), and DJ-1 (PARK7) [5, 6]. In addition, as an important regulator at posttranscriptional level, several miRNAs have been discovered to be involved in PD pathogenesis via regulating PD-associated gene expression. For example, miR-7 and miR-153 are recently described to regulate endogenous synuclein levels; inhibition of *α*-synuclein expression by miR-7 protects against oxidative stress-mediated cell death [7, 8]; several studies suggest that the role of LRRK2 in the pathogenesis of PD is mediated through the miRNA pathway [9]. 

Dorsal motor nucleus of the vagus (DMNV) and inferior olivary nucleus (ION) are two brainstem regions which may be damaged early in the course of PD [10, 11]. However, the molecular mechanism of these two regions is not well understood for PD. In this study, we aimed to compare the gene expression profiles of DMNV and ION from PD patients with that of controls using oligonucleotide microarray. Microarray experiments can simultaneously measure the expression levels of thousands of genes, generating huge amounts of data, [12] and have been applied to identify molecular markers of PD in several studies [13, 14]. In addition, the related miRNAs that were mapped to their target differentially expressed genes (DEGs) were also analyzed by bioinformatics methods to reveal the regulatory mechanism. 

## 2. Materials and Methods

### 2.1. Microarray Data

Gene expression data set GSE19587 [15] was downloaded from Gene Expression Omnibus [16]. It contained two groups of gene expression profiles: 6 DMNV samples from patients with PD and 5 from controls; 6 ION samples from patients with PD and 5 from controls. The platform was GPL571 [HG-U133A_2] Affymetrix Human Genome U133A 2.0 Array. Probe annotation files were also acquired.

### 2.2. Preprocessing and Differential Analysis

Raw data were converted into recognizable format with package affy of *R*, and missing values were then imputed [17]. After data normalization with median method [18], differential analysis between PD and control was performed using package limma [19] for DMNV and ION, respectively. |log⁡(fold  change)FC | >1 and *P* < 0.05 were set as the cut-offs to screen out DEGs.

### 2.3. Gene Ontology (GO) Functional Enrichment Analysis of DEGs

In order to identify disturbed biological functions in PD, GO functional enrichment analysis was performed for DEGs in DMNV and ION using DAVID with a threshold of *P* < 0.05 [20]. DAVID is the Database for Annotation, Visualization and Integrated Discovery, providing a comprehensive set of functional annotation tools for the investigation of the biological meaning behind large list of genes.

### 2.4. Comparison of DEGs between DMNV and ION

Common DEGs from the two regions of the medulla (DMNV and ION) were obtained using package Venn of *R*.

### 2.5. Establishing Interaction Network between Common DEGs and miRNAs and Functional Enrichment Analysis for DEGs in Network

miRNAs which targeted the common DEGs were retrieved with WebGestalt [21, 22]. For multiple testing correction, the Benjamini-Hochberg (BH) approach was used [23], and miRNAs with BH-adjusted *P* < 0.05 (false discovery rate (FDR) < 0.05) were selected. The regulatory network between DEGs and miRNAs and interactions between DEGs were then visualized with Cytoscape. In addition, GO functional enrichment analysis was applied on the genes in the network via DAVID with a threshold of *P* < 0.05. 

## 3. Results

### 3.1. DEGs in DMNV and ION

**Figure 1:**
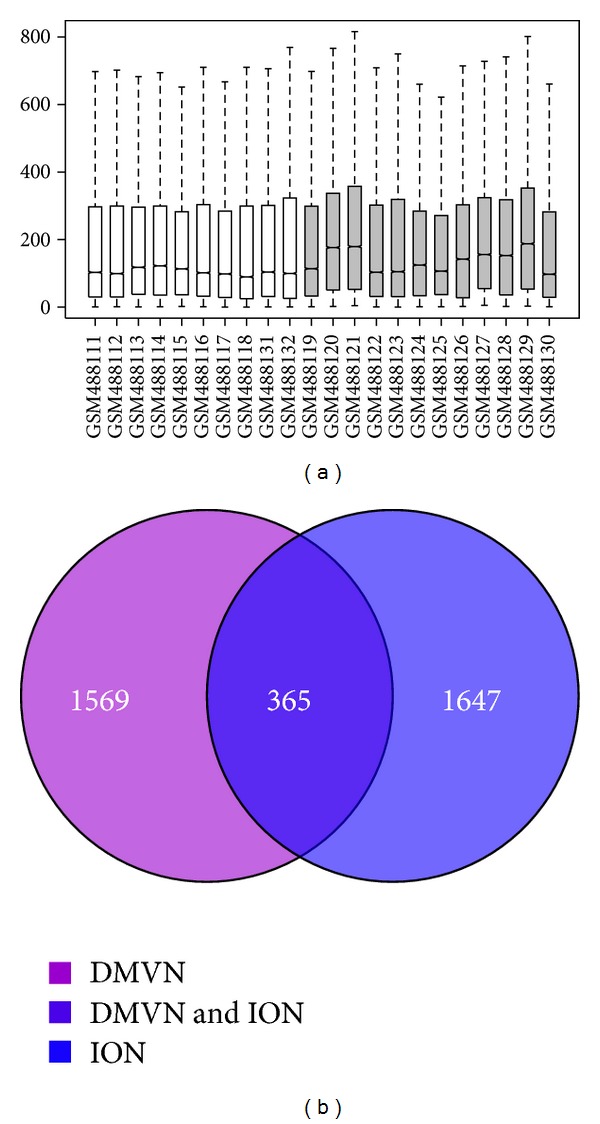
Box plot for normalized gene expression data. The medians (black lines) are almost at the same level, indicating a good performance of normalization (a). Venn diagram of differentially expressed genes identified from dorsal motor nucleus of the vagus (DMNV) and inferior olivary nucleus (ION) of PD (b).

After gene expression data normalization (Figure 1(a)), 1569 (DMNV) and 1647 (ION) DEGs for PD were screened by comparison between the samples from PD patients and controls. As shown in Figure 1(b), 385 common DEGs presented both in DMNV and ION of PD patients were extracted from these identified DEGs.

### 3.2. Functional Enrichment Analysis Results

**Figure 2:**
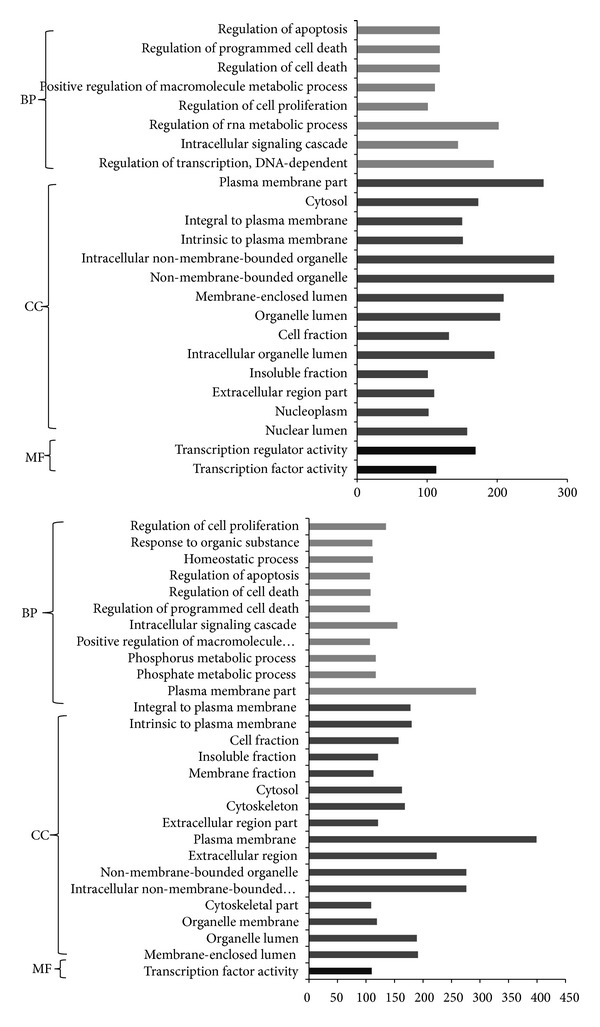
Overrepresented gene ontology terms for differentially expressed genes from dorsal motor nucleus of the vagus (DMNV, above) and inferior olivary nucleus (ION, below). BP: biological process; CC: cellular component; MF: molecular function.

Significantly overrepresented GO terms were revealed by using DAVID. A total of 24 and 28 terms were disclosed for DEGs in DMNV and ION, respectively (Figure 2), in which DEGs from DMNV and ION seemed to share similar biological processes, such as regulation of cell proliferation, positive regulation of macromolecule metabolic process, regulation of apoptosis, and so on.

### 3.3. miRNAs and Gene Regulatory Network

**Figure 3:**
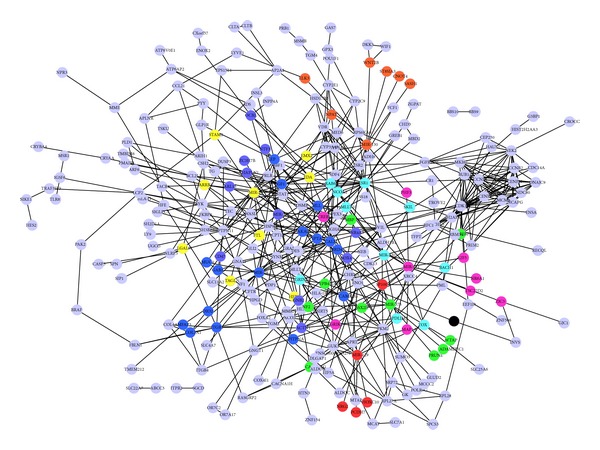
The integrated DEGs-miRNAs regulatory and DEGs-DEGs interaction network. miRNAs and their target genes shared the same color. DEGs: differentially expressed gene.

A total of 8 relevant clusters of miRNAs were retrieved with WebGestalt for the common DEGs (Table 1). Then the miRNAs-DEGs regulatory network and DEGs-DEGs interaction network were visualized with Cytoscape (Figure 3). Functional annotation was applied on the genes in the network, and 19 GO terms were revealed (Table 2), among which regulation of apoptosis was the most significant one.

## 4. Discussion

In the present study, we identified 1569 and 1647 DEGs in DMNV and ION, respectively through the comparative analysis of transcriptome between PD and controls. Also, we found 365 common DEGs presented in both regions, as well as 8 related miRNAs which targeted these common DEGs. Finally, we constructed an integrated network, including the DEGs-DEGs interactions, and the DEGs-miRNA regulatory network consisting of 8 miRNAs (MIR-22, MIR-181, MIR-129, MIR-29, MIR-373, MIR-330, MIR-130, and MIR-374) and their target common DEGs. 

Apoptosis plays a critical role in the pathogenesis of PD [24, 25]. In present study, many DEGs involved in apoptosis were found in the two regions of the medulla. Functional enrichment analysis of DEGs indicated that regulation of apoptosis was the one of the top 3 biological processes for both groups of DEGs. Moreover, thirty-one DEGs in the regulatory network were also enriched in regulation of apoptosis (the top one GO term). It has been reported that some DEGs (e.g., VDR, NTF3, CREB1, and IGF1) within the apoptosis pathway may contribute to the pathogenesis of PD according to the previous literature. Vitamin D has been demonstrated to regulate cell proliferation in the developing brain [26], and vitamin D deficiency alters dopamine turnover in the forebrain and dopamine-mediated movement, resulting in high risk for PD [27, 28]. Vitamin D receptor (VDR) is the primary mediator of vitamin D's biological actions; that is, vitamin D is first converted to the active metabolite 1,25-dihydroxy vitamin D3. Upon binding to 1,25-dihydroxy vitamin D3, VDR is activated and interacts with vitamin D responsive elements in the promoters of vitamin D target genes to regulate their expression [29, 30]. Moreover, several studies also report an association between VDR polymorphism and PD [31, 32]. Neurotrophin 3 (NTF3) is a member of the neurotrophin family, which controls the survival and differentiation of mammalian neurons. The delivery of NTFs has been postulated as a therapy for neurodegenerative disorders like PD [33, 34]. As a member of the leucine zipper family of DNA binding proteins, CREB1 (cAMP responsive element binding protein 1) may play an important role in the dopaminergic activation of c-fos in the striatum, and the lacking of a CREB1-induced transcription cascade may contribute to long-lasting psychomotor disorders in PD [35]. Ebert et al. report that human neural progenitor cells overexpressing IGF1 (insulin-like growth factor 1) can protect dopamine neurons and restore function in a rat model of PD [36]. 

miRNAs are important regulators participating in many physiological processes and thus become therapeutic targets for diseases, such as cancers and neurodegenerative diseases [37]. To discover potential molecular targets, miRNAs interacting with DEGs were retrieved in the present study and regulatory network was also constructed. Aberrant expression of miR-22 has been identified in multiple human diseases [38]. It shows low expressions in PD blood samples, and it can be used to distinguish nontreated PD from healthy subjects [39]. Ferritin light polypeptide (FTL) is regulated by miR-22. FTL is the light subunit of the ferritin protein, which is the major intracellular iron storage protein. Previous studies have indicated that disturbances in brain iron homeostasis may contribute to the pathogenesis of PD [40, 41]. Thus, we suppose that FTL and miR-22 are worthy of further investigations to disclose their specific roles in PD. miR-181 is implicated in apoptosis. Downregulation of miR-181 permits Bcl-2 to remain at a high level without posttranscriptional repression, which eventually leads to the gain in neuronal survival [42] and may decrease the incidence of PD. DEGs regulated by this miRNA included CREB1 and estrogen receptor 1 (ESR1). The expression level of miR-29 can also be used to distinguish nontreated PD from healthy subjects [39]. IGF1 and calcium/calmodulin-dependent protein kinase II gamma (CAMK2G) are regulated by this miRNA. CAMK2G links endoplasmic reticulum stress with Fas and mitochondrial apoptosis pathways [43]. Inhibitors of CAMK2G may be useful in preventing apoptosis in pathological settings and even treat diseases like PD.

Overall, our study provides an integrated network insight into the pathogenesis of PD and offers potential therapeutic targets for controlling the disease. Although previous studies have implicated that brainstem regions including DMNV and ION are relatively unaffected and not obligatory trigger sites of PD [10, 44, 45], the genes in DMNV and ION are demonstrated to be associated with neuron death in our study, and thus deep experiment researches in these regions are still needed. 

## Figures and Tables

**Table 1:** Relevant miRNAs for the 365 common differentially expressed genes.

miRNA	DB_Num	Parameters
hsa_GGCAGCT, MIR-22	DB_ID:780	*O* = 13; raw*P* = 8.79*e* − 08; adj*P* = 1.76*e* − 06
hsa_TGAATGT, MIR-181A, MIR-181B, MIR-181C, MIR-181D	DB_ID:669	*O* = 16; raw*P* = 3.73*e* − 06; adj*P* = 2.56*e* − 05
hsa_GCAAAAA, MIR-129	DB_ID:798	*O* = 10; raw*P* = 3.84*e* − 06; adj*P* = 2.56*e* − 05
hsa_TGGTGCT, MIR-29A, MIR-29B, MIR-29C	DB_ID:671	*O* = 16; raw*P* = 9.23*e* − 06; adj*P* = 4.61*e* − 05
hsa_TTTTGAG, MIR-373	DB_ID:844	*O* = 10; raw*P* = 2.08*e* − 05; adj*P* = 8.32*e* − 05
hsa_TGCTTTG, MIR-330	DB_ID:843	*O* = 12; raw*P* = 2.76*e* − 05; adj*P* = 9.20*e* − 05
hsa_TTGCACT, MIR-130A, MIR-130B	DB_ID:676	*O* = 13; raw*P* = 3.80*e* − 05; adj*P* = 0.0001
hsa_TATTATA, MIR-374	DB_ID:727	*O* = 10; raw*P* = 0.0002; adj*P* = 0.0005

DB_Num: number assigned by the database; *O*: number of differentially expressed genes regulated by the miRNA; raw*P*: initial *P* value calculated according to the hypergeometric distribution; adj*P*: *P* value after adjusted with the Benjamini-Hochberg correction method.

**Table 2:** Overrepresented GO terms in genes from the regulatory network.

Term	Count of DEGs	*P* value
GO:0042981~regulation of apoptosis	31	0.0026939
GO:0043067~regulation of programmed cell death	31	0.0031183
GO:0010941~regulation of cell death	31	0.0032783
GO:0070271~protein complex biogenesis	22	0.0034321
GO:0006461~protein complex assembly	22	0.0034321
GO:0043085~positive regulation of catalytic activity	22	0.0048116
GO:0065003~macromolecular complex assembly	26	0.0054608
GO:0048878~chemical homeostasis	21	0.0082118
GO:0007267~cell-cell signaling	23	0.0117819
GO:0043933~macromolecular complex subunit organization	26	0.0119356
GO:0042127~regulation of cell proliferation	28	0.0126938
GO:0042592~homeostatic process	27	0.0128669
GO:0007049~cell cycle	27	0.0185901
GO:0022402~cell cycle process	21	0.0222268
GO:0006357~regulation of transcription from RNA polymerase II promoter	25	0.027631
GO:0010604~positive regulation of macromolecule metabolic process	28	0.0335407
GO:0031328~positive regulation of cellular biosynthetic process	23	0.0437151
GO:0010557~positive regulation of macromolecule biosynthetic process	22	0.0482295
GO:0009891~positive regulation of biosynthetic process	23	0.049702

GO: gene ontology; DEGs: differentially expressed genes.
